# Integrating Environmental and Human Health Databases in the Great Lakes Basin: Themes, Challenges and Future Directions

**DOI:** 10.3390/ijerph120403600

**Published:** 2015-03-31

**Authors:** Kate L. Bassil, Margaret Sanborn, Russ Lopez, Peter Orris

**Affiliations:** 1 Toronto Public Health, Toronto, ON M5B 1W2, Canada; 2 Dalla Lana School of Public Health, University of Toronto, Toronto, ON M5T 3M7, Canada; 3 Department of Family Medicine, McMaster University, Hamilton, ON L8P 0A1, Canada; E-Mail: msanborn@sbghc.on.ca; 4 Dukakis Center for Urban and Regional Policy, Northeastern University, Boston, MA 02115, USA; E-Mail: rptlopez@gmail.com; 5 School of Public Health, University of Illinois at Chicago, Chicago, IL 60612, USA; E-Mail: porris@uic.edu

**Keywords:** environmental hazards, health outcomes, surveillance, database integration

## Abstract

Many government, academic and research institutions collect environmental data that are relevant to understanding the relationship between environmental exposures and human health. Integrating these data with health outcome data presents new challenges that are important to consider to improve our effective use of environmental health information. Our objective was to identify the common themes related to the integration of environmental and health data, and suggest ways to address the challenges and make progress toward more effective use of data already collected, to further our understanding of environmental health associations in the Great Lakes region. Environmental and human health databases were identified and reviewed using literature searches and a series of one-on-one and group expert consultations. Databases identified were predominantly environmental stressors databases, with fewer found for health outcomes and human exposure. Nine themes or factors that impact integration were identified: data availability, accessibility, harmonization, stakeholder collaboration, policy and strategic alignment, resource adequacy, environmental health indicators, and data exchange networks. The use and cost effectiveness of data currently collected could be improved by strategic changes to data collection and access systems to provide better opportunities to identify and study environmental exposures that may impact human health.

## 1. Introduction

There are vast amounts of available data routinely collected by governments and academic and research institutions that are relevant to environmental health. There is a growing need to integrate environmental and human health data to enable decision makers to make more informed protection and restoration decisions related to ecosystems and public health, with the ultimate goal of reducing the environmental burden of disease. However, integrating these datasets so the information is more accessible and usable poses several challenges. In 2012, the International Joint Commission’s Health Professional Advisory Board (HPAB) initiated a review of existing environmental and human health datasets in the Great Lakes area to identify opportunities and challenges for integrating these data.

The Great Lakes are the world’s largest freshwater ecosystem, with a heavily populated surrounding basin. Significant pollution of the Great Lakes can expose the 35 million basin residents to serious health problems while imposing recreational restrictions and economic losses [1]. To mitigate this, the Great Lakes Water Quality Agreement between Canada and the United States aims to restore and maintain the chemical, physical and biological integrity of the Great Lakes and includes several specific objectives to accomplish this [1]. However, one of the important challenges that environmental health practitioners and policy-makers face in addressing these issues is an incomplete understanding of the specific health impacts of the contamination of the Great Lakes Basin and their magnitude. A barrier to attaining this understanding is the lack of integrated environmental and human health currently collected data.

There are several benefits to integrating environmental and human health data. For example, when the etiology of a disease is unknown but environmental causes are hypothesized, linking human health and exposure data can facilitate the examination of potential associations [2,3,4]. For example, in order to test the hypothesis that living near an area with persistent organic pollutants can increase exposure and subsequent risk of developing non-communicable diseases, such as heart diseases, stroke, and diabetes, researchers have linked hospitalization discharge rates with the concentration of persistent organic pollutants based on zip code in New York State [5,6,7,8,9]. Similarly, researchers have integrated asthma emergency department visits/hospitalization discharge data with air pollution data (e.g., concentrations of particulate matter) based on zip code/area code to assess the association between air quality and asthma episodes among adults and children in Canada and the US [10,11,12,13,14].

If the association between human health and the environment has already been established, linking environmental stressors, human exposure and human health outcome data can help policy makers monitor trends and identify opportunities for intervention [2,3,4]. For example, in Oregon, based on the findings from the state-wide Environmental Public Health Tracking Program, the levels of arsenic in more than 100 residential wells in the Sutherlin Valley were found to exceed healthy drinking water standards [15]. Since long-term exposure to arsenic is known to have a negative impact on reproductive organs, the heart, skin and nervous system, the state of Oregon passed legislation in 2009 to make testing arsenic in private wells mandatory, as a result of the study’s findings [15].

Integrating environmental and health data can also provide opportunities to engage and inform the public about potential associations and ways to mitigate the effects [3]. For example, research conducted as part of the Florida Environmental Public Health Tracking Program found that women of child-bearing age in certain counties consumed more fish than their counterparts in other areas of the US and had higher hair mercury levels than participants in the study who did not consume fish [16]. As a result, educational wallet cards were developed and distributed to the public about the types and amount of fish to eat to avoid unsafe amounts of mercury [16].

There are several examples of the successful integration of environmental and human health data that range from large national programs to smaller local research studies. Many of these were initiated following the publication of the landmark Pew Environmental Health Commission report “America’s Environmental Health Gap: Why the Country Needs a Nationwide Health Tracking System”, which highlighted the environmental health data gaps in the United States and recommended the development of a nation-wide environmental health tracking network [17]. In response to these recommendations, the Centers for Diseases Control and Prevention (CDC) initiated the development of the National Environmental Public Health Tracking program to facilitate the integration and dissemination of environmental hazard, human exposure and health outcome surveillance data across the US [18]. Internationally, the World Health Organization’s Regional Office for Europe has also developed a similar environmental public health tracking system, the Environment and Health Information System, to integrate environmental and health data across the European Union [19].

While Canada does not currently have a national environmental health tracking program, there have been efforts to develop environmental health surveillance datasets and capacity [20,21]. In 2001, the Federal/Provincial/Territorial Environmental and Occupational Health Surveillance Working Group prepared a detailed inventory of environmental and occupational datasets in Canada [22]. Similarly, the First Nations Environmental Health Innovation Network developed an inventory of environmental health datasets related to the First Nations communities in Canada [23]. Statistics Canada convened an environmental-human health expert panel to identify potential opportunities to use national statistics to link human health and the environment [21].

There are fewer examples of binational collaborations to integrate environmental and health data, likely due to the added complexity of cross-border data sharing. However, one example is the Border Air Quality Study that is investigating the association between exposure to air pollutants and several health outcomes in the Georgia Basin Puget Sound region that spans the border between British Columbia in Canada and Washington in the US [24].

The purpose of this review is to describe the existing databases relevant to environmental health in the Great Lakes Basin and identify opportunities and challenges for integrating these data to enhance the capacity for evidence-informed environmental health decision-making.

## 2. Materials and Methods

The identification and review of environmental health databases was conducted using both a literature search and series of expert consultations.

### 2.1. Literature Review

A systematic literature search was conducted using the databases, PubMed and ScienceDirect to identify any environmental and human health databases relevant to the Great Lakes Basin. The search was limited to English publications and included a broad range of search terms in an effort to capture a comprehensive list of datasets. Supplementary Material, Table S1 provides further details of the search formats applied.

In addition to peer-reviewed literature, websites of key government and environmental health agencies were also searched to identify additional datasets. Demographic and spatial datasets were also included given their relevance to environmental health associations. The list of websites and agencies searched can be found in Supplementary Material, Table S2.

Databases were identified and organized by type of data collected in the following categories:

1. *Environmental stressors*: Any physical, chemical, or biological entity that can cause an adverse impact [25]. Examples include databases that capture water quality, air quality, soil/land quality, concentrations of toxins in fish/biota, industrial run-off, waste water, nutrients run off, and pathogens.
2. *Human exposure*: Human exposure data describe the extent to which an individual may encounter environmental hazards through ingestion, inhalation, dermal exposure, or other pathways.
3. *Health outcome*: Human health outcome data describe the burden of illnesses in a population, which includes mortality, morbidity as well as quality of life measures such as Quality Adjusted Life Year (QALY) and Disability Adjusted Life Year (DALY).

An inventory of the databases was developed that included information about the data included in the database, the organization responsible for managing/collecting the data, and its geographical coverage. An assessment of the inventory was conducted to highlight some of the key characteristics of existing environmental and health datasets in the Great Lakes area, including data availability, accessibility and emphasis on the needs of the vulnerable populations in the Great Lakes Basin.

### 2.2. Expert Consultations

A series of consultations with environmental health experts and Great Lakes researchers in Canada and the United States was conducted in January and February 2013 to identify databases and examine the opportunities and challenges for integrating environmental health data in the Great Lakes Basin. Over a four-week period, consultations with experts were conducted through a combination of in-person meetings, 30–60 min telephone calls and/or e-mail correspondence. Common themes from the expert consultation interviews were extracted to capture shared viewpoints and experiences related to data collection and use, data gaps, and other challenges related to integrating environmental and health data.

## 3. Results

### 3.1. Identification of Environment and Health Databases

**Figure 1:**
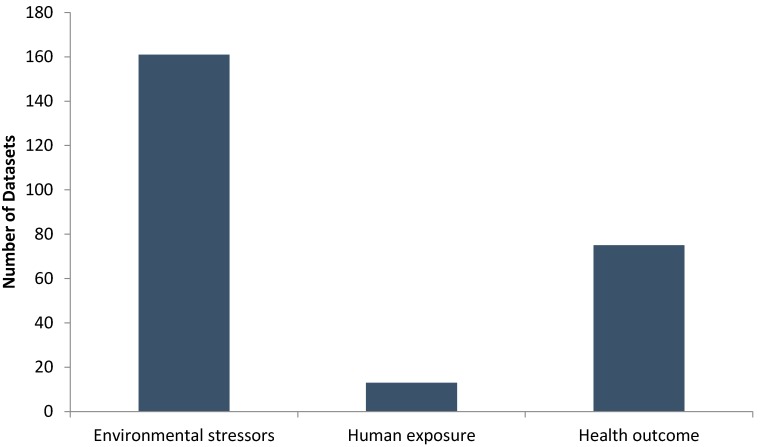
Distribution of identified environment and health databases.

Over 250 health and environmental datasets were identified and reviewed in the search. The majority of these represent environmental stressors data. A smaller number of databases were found that represent human health outcomes, and the fewest from the human exposure data category. The total number of databases in each of these three categories is illustrated in Figure 1. The inventory of databases can be found in a technical report of this work [26].

Nearly half of the datasets represent environmental stressors data (*n*=161). These include databases that capture air quality, water quality, soil quality, fish/biota contamination, harmful algae blooms, food recalls, and smoke density, at both the national and regional level. National and provincial/state-wide environmental agencies collect a large portion of these datasets. This category also includes databases that represent mandatory reporting by industrial facilities in both Canada and the United States. In Canada, the National Pollutant Release Inventory (NPRI) is a legislated inventory that tracks pollutant releases, and disposal reported by industrial facilities [27]. Similarly, the US Environmental Protection Agency (EPA) Toxic Release Inventory (TRI) is the key source of environmental stressors information in the United States that contains pollutant disposal and chemical releases data from over 23,000 industrial facilities across the United States [28].

Health outcome data represented the next largest group of databases (*n* = 75), and spanned a range of health outcomes, most often captured in administrative databases (e.g., vital statistics, hospital admissions, disease registries, physician/emergency department visits). Large-scale national surveys are also a key source of health outcome data in both Canada and the United States.

Human exposure databases represent the smallest group of datasets identified in the search (*n* = 13). These datasets can be broadly categorized into results from biomonitoring studies, exposure estimates, and survey findings. While biomonitoring studies are often considered the “gold standard” for assessing exposure to contaminants they are typically costly to conduct. Despite this, there are a few examples of biomonitoring studies including the Canadian Health Measure Survey (CHMS), Maternal-Infant Research on Environmental Chemicals (MIREC), and the First Nations Biomonitoring Initiative [29] in Canada, and the National Health and Nutrition Examination Survey (NHANES) and the National Children Study (NCS) in the US. However, samples are typically collected at a selection of collection sites. Though the data are sampled and weighted to give a demographically representative sample, the small number of sites makes estimating the Great Lakes Basin population’s level of specific exposure to environmental hazards a challenge. Two exceptions to this are the National Human Exposure Assessment Survey (NHEXAS) Phase 1 field study and the Contaminants in Human Tissues Survey that have sampled specifically within the Great Lakes Basin.

Overall, the literature search identified that there are vast data available that represent characteristics of the environment and human health. However, these databases are not currently integrated and most do not focus on the Great Lakes Basin specifically, despite the somewhat unique environmental health issues relevant to this area. The exception to this is research studies that have been conducted in the region that integrated environmental and health data to examine specific associations. Many of these existing studies have focused primarily on linking reproductive outcomes (e.g., low birth weight, decreased head circumference, and earlier gestational age) with the consumption of contaminated Great Lakes fish [30,31,32]. Follow-up studies of these infants report associations with neurobehavioural deficits including poorer motor reflex and neuromuscular functioning and depressed responsiveness [33]. Other health outcomes that have been found to be associated with contaminants in the Great Lakes include diabetes, memory and learning impairments, and uterine fibroids [34,35,36,37]. Additionally, there have been a few studies with a specific focus on a Great Lakes Areas of Concern that have integrated health outcome data with water and air pollution data. For example, a study in Windsor linked respiratory hospitalization data with several air pollutants and found several statistically significant associations [38].

### 3.2. Challenges and Opportunities for Integrating Environment and Health Databases

There were nine key factors that impact integration identified from the literature search and expert consultations. These include: data availability, accessibility, harmonization, stakeholder collaboration, policy and strategic alignment, resource adequacy, environmental-human health indicators and data exchange networks.

#### 3.2.1. Data Availability

Findings from the expert consultations noted that data availability is one of the key barriers to integrating environmental and health data, particularly for the Great Lakes Basin. Data availability includes both the generalizability and specificity of existing datasets. Ideally, the available datasets should be generalizable to represent the entire Great Lakes Basin, but should also allow analysis of sub-regions or specific parts of the Great Lakes Basin. However, this review found that most of the existing environment and health datasets in Canada and the United States are typically collected at the national, provincial or state level. As such, the existing datasets typically cannot always be generalized to the population of the Great Lakes Basin. Additionally, the characteristics and scope of the existing environmental datasets are often defined by the environmental regulation, compliance and enforcement of specific jurisdictions they represent, which may not be for the purpose of environmental health surveillance in the Great Lakes Basin. For example, as mentioned earlier, both TRI and NPRI are inventories of emissions released by industrial facilities. However, only those industries that meet a specific reporting threshold (based on chemicals manufactured, processed, or used) are required to report on an annual basis [39,40]. There are minimal data collected on facilities that discharge contaminants below the reporting threshold for NPRI and TRI, although many of these smaller industries emit pollutants that have been associated with adverse health outcomes. The ChemTRAC program in Toronto is an example of a program that assesses emissions from smaller to medium sized organizations as the reporting thresholds are lower than those used for the NPRI (www.toronto.ca/chemtrac).

This review also found a general lack of data about vulnerable populations in the Great Lakes Basin including children, pregnant women, residents of the Areas of Concern and First Nations/Tribal communities. Most of the datasets that relate to these vulnerable populations are captured through standalone research studies [31,32,41,42]. To date, the Contaminants in Human Tissues Survey is the only large-scale dataset that specifically captures the First Nations communities’ exposure to environmental hazards in the Great Lakes region [43].

#### 3.2.2. Data Accessibility

Health and environmental datasets relevant to the Great Lakes Basin are managed and stored separately by various state, provincial, national environmental and health agencies in Canada and the United States, each with their own data access policies and procedures. There are also differences in the legislations governing the collection and release of data, particularly heath data, at the municipal/state/provincial/federal level. Additionally, the responsible organizations that monitor and collect these data vary across the different Great Lakes states and provinces. For example, in Ohio, the Department of Natural Resources is responsible for monitoring water quality data, while in Illinois, the water quality data is collected by both the Illinois Environmental Protection Agency and Illinois State Water Survey. This variation in the way data are collected and housed across provinces and states, and the lack of uniformity in policies for accessing these data pose a particular challenge to integrating environment and health data for the Great Lakes region.

#### 3.2.3. Data Harmonization

This review found that in the absence of a common reporting standard for Great Lakes environmental and health data, there is a varying data structure and spatial scale across datatsets that is a significant barrier to integration. However, there have been recent efforts to harmonize datasets in some sectors. For example, the EPA’s Environmental Information Exchange Network is a web-based information sharing initiative that provides state environmental agencies easy access to federal environmental monitoring data and to the environmental data of other states using standardized data formats. This Exchange Network has been used to develop a comprehensive and harmonized source of water quality data for several US states.

#### 3.2.4. Stakeholder Collaboration

Findings from the expert consultations suggest that there has been a growing interest in data integration and knowledge exchange among health and environmental organizations in Canada and United States. For example, in the late 1980s the Binational Executive Committee (BEC), comprised of key environmental stakeholders from Canada and United States, was formed to carry out activities and programs to fulfill the requirements set out by the Great Lakes Water Quality Agreement [44]. The Great Lakes Interagency Task Force and Regional Collaboration were established in 2004 by an executive order to improve federal coordination and harmonize goals. This effort was implemented to a great extent by the Great Lakes Restoration Initiative. More recently, the Cooperative Science and Monitoring Initiative was formalized to institutionalize and coordinate environmental related monitoring and research field activities in the Great Lakes region [44]. However, the coordinated initiatives in the Great Lakes Basin to date have a strong focus on environmental surveillance and monitoring. While the Chemical Management Plan in Canada and the CDC National Environmental Public Heath Tracking System have forged partnerships between environmental and health stakeholders at the national level, there have been limited knowledge exchange initiatives and cooperatives efforts, especially among health stakeholders in the Great Lakes area.

#### 3.2.5. Policy and Strategic Alignment

In the absence of a strong collaborative effort among the health and the environmental sectors in Canada and the United States, surveillance initiatives in the Great Lakes area have largely been developed independently and without inter-agency collaboration. Consequently, the availability and structure of environmental health in Canada and the United States varies according to the needs and resource capacity of the jurisdictions. Additionally, most of the strategies and policies related to the Great Lakes region, such as the Great Lakes Binational Toxic Strategy and the Great Lakes Regional Collaboration Strategy have focused on ecosystem health and not human health. There is a need for improved alignment of environmental and health surveillance priorities in the Great Lakes Basin in order to identify key areas of focus for database integration.

#### 3.2.6. Resource Adequacy

Currently the existing surveillance activities in the Great Lakes Basin have placed a strong emphasis on the health of the Great Lakes ecosystem, with less focus on human health. Many of the Great Lakes monitoring initiatives, such as the Great Lakes Information Network and Great Lakes Observing Systems, are funded and supported by environmental organizations such as the Great Lakes Commission and Coastal Services Center of the National Oceanic and Atmospheric Administration.

In Canada, the Ontario Ministry of Natural Resources, Ministry of the Environment and Climate Change, Environment Canada and Department of Fisheries and Oceans fund a large portion of the surveillance activities in the Great Lakes region. Although Health Canada and the Ontario Ministry of Health and Long Term Care have been involved in supporting a few Great Lakes surveillance and monitoring activities, such as the Great Lakes Human Effects Programs, Great Lakes Border Health Initiatives, Contaminants in Human Tissues Survey, in comparison to the environment sector, the health sector has played a smaller role in supporting surveillance activities in the Great Lakes Region.

#### 3.2.7. Environmental-Human Health Indicators

Of the 77 indicators established under the United States-Canadian State of Lakes Ecosystem Conference (SOLEC) to monitor the health of Great Lakes Basin, relatively few are related to human health. To date, there are five human impact SOLEC indicators: Beach Advisories, Cladophora, Drinking Water Quality, Fish Consumption Restrictions, and Harmful Algal Bloom [45,46,47]. However, although the SOLEC indicators do focus on hazards which may have a human health impact, they are generally not directly quantified in terms of human health risk and presently do not directly measure any human health outcomes. As a result, the Health Professionals Advisory Board of the International Joint Commission is working towards identifying and defining potential human health indicators that may reflect progress towards protecting and restoring the waters of the Great Lakes.

#### 3.2.8. Data Exchange Network

This review found that while there are data exchange initiatives that have been developed in the United States, similar data exchange initiatives have not yet been established to the same extent in Canada. For example, the US EPA and the CDC have placed significant resources into developing tools and systems to facilitate the sharing of environmental and health data across the United States. The previously mentioned EPA Environmental Information Exchange Network is a good example of this; it aims to provide a centralized technology platform that facilitates the sharing and use of environmental data [48]. The Environmental Information Exchange Network enables stakeholders to connect and exchange data with other partners securely online using a standardized data structure. Furthermore, to resolve any potential data accessibility concerns, partners worked together to develop data sharing agreements at the initial phase of the project [48]. Through this network, initiatives such as the GLENDA Query System, Great Lakes Node project and the Geo-Exchange with Region 5 and Wisconsin Department of Natural Resources have been established to facilitate environmental data exchange within the Great Lakes Basin.

The CDC initiated the development of the National Environmental Public Health Tracking program. The purpose of this program is to facilitate the integration and dissemination of environmental hazard human exposure and health effects surveillance across the United States [49]. Currently, the program provides grants to 23 states and one city as a way to facilitate the development and implementation of state-wide and local environmental public health tracking network [50]. In addition, as part of the Peer Fellowship program, the National Environmental Public Health Tracking program also provides 13 states with the necessary tools and training to build their own local tracking networks [50]. The CDC’s National Environmental Public Health Tracking System has been used in a wide range of applications, including public education, policy development, targeted prevention, and identification of at risk communities. For example, in California, the tracking system has been used to identify the increase in preterm rates in California County, to inform policy about heat wave preparedness and to develop a traffic tool for city planning [51].

Currently there are not comparable systems in Canada. However, Public Health Ontario, a provincial health agency, is examining the feasibility of developing an environmental health tracking system for Ontario that could be used by public health practitioners.

## 4. Discussion

Currently neither health nor environmental exposure data are collected in a way that easily allows examination of associations in the Great Lakes ecosystem. The existing national health surveys, although designed to be nationally representative, cannot be used to ascertain environmental health risks for the Great Lakes Basin and compare them to the rest of the two countries. The HPAB has developed the following set of recommendations to address these basic concerns:

*Recommendation #1*: That governments actively engage with public health practitioners, academic researchers, and community groups that represent vulnerable communities in the Great Lakes Basin to identify priority areas for data integration. This report has illustrated the vast data sources that could potentially be integrated, however, it will be important for the data users to identify knowledge gaps and priorities for surveillance that the integration process could address.

*Recommendation #2*: That governments explore the potential of building onto databases already in place (e.g., Great Lakes Observing System) and/or projects that have already developed exposure estimate techniques and models for similar purposes (e.g., CAREX Canada). These existing projects have the potential to be extended by sampling in the Great Lakes Basin to develop a dataset that is more representative of the Great Lakes Basin population.

*Recommendation #3*: That the two governments establish a cooperative data management system for human health and environmental exposure for the Great Lakes Basin, providing a framework for data access for researchers and the general public. This could be achieved by funding a reference to the IJC, or enhancing existing capacities within governments such as the IJC’s Great Lakes Regional Office or the Great Lakes Observing System, to create a binational data centre. Such a centre would coordinate and collect health and exposure surveillance information relevant to the Great Lakes Basin in such a way as to permit ongoing monitoring, academic access for particular studies, and public access to the data where appropriate.

*Recommendation #4*: That governments improve the quality and usability of data by making funding proportional to the quality, accessibility and harmonizability of data collected in the Great Lakes. This should be done in conjunction with a basin-wide initiative to harmonize existing datasets and develop agreements on their joint transboundary use.

*Recommendation #5*: That the National Health and Nutrition Examination Survey (NHANES) and Canadian Health Measures Survey (CHMS) include an oversampling for the Great Lakes region that will allow comparisons to the rest of the two countries. This is necessary because neither survey is constructed to provide information on which health outcomes occur more or less often in the Great Lakes Basin.

*Recommendation #6*: That the parties expand the suite of human health indicators that provide meaningful information on the state of human health related to the Great Lakes environment. Ideally these should move from the current exposure-based indicators into health outcome indicators. The indicators would then guide decisions about priority areas for better data collection and monitoring of human exposures and associated health impacts.

*Recommendation #7*: Biomonitoring resources should be focussed on three vulnerable sub populations: young children, pregnant women and First Nations/Tribal groups. Health assessments need to accompany biomonitoring information to make them useful for understanding exposure-health associations.

## 5. Conclusions

The use and cost effectiveness of data currently collected could be improved by strategic changes to data collection and access systems to provide better opportunities to identify and study environmental exposures that may impact health. Ultimately, integrated data will enable decision-makers to make better informed protection and restoration decisions related to ecosystems and public health.

## Supplementary Files

Supplementary File 1
