# Supplementary material for: Integrating Environmental and Human Health Databases in the Great Lakes Basin: Themes, Challenges and Future Directions
Source: Int J Environ Res Public Health. 2015 Mar 31;12(4):3600–14. doi: 10.3390/ijerph120403600 (PMC4410205; doi:10.3390/ijerph120403600)
Supplement: Supplementary File 1 [file ijerph-12-03600-s001.pdf]

Supplementary Information

Integrating Environmental and Human Health Databases in the Great Lakes Basin: Themes, Challenges and Future Directions

---

Table of Contents:

Table S1. Search Terms Used in Article Database Searches.

Great Lakes AND (health OR health outcomes) AND (environmental stressors OR environmental hazards OR environment OR datasets OR water quality OR air pollution OR air quality OR monitoring OR surveillance OR environmental tracking OR environmental health indicators OR area of concern OR public health tracking OR survey OR toxic OR toxins OR harmful algal blooms OR run-offs OR farm management OR hazardous waste OR contamination OR biomonitoring OR vulnerable populations OR Aboriginal OR Tribal OR children OR pregnant women OR fetus OR diet OR fish consumption OR surface water OR ground water OR stream OR river OR wetlands OR drinking water OR well OR sport fish OR discharge)
Lake Ontario AND (health OR health outcomes) AND (environmental stressors OR environmental hazards OR environment OR datasets OR water quality OR air pollution OR air quality OR monitoring OR surveillance OR environmental tracking OR environmental health indicators OR area of concern OR public health tracking OR survey OR toxic OR toxins OR harmful algal blooms OR run-offs OR farm management OR hazardous waste OR contamination OR biomonitoring OR vulnerable populations OR Aboriginal OR Tribal OR children OR pregnant women OR fetus OR diet OR fish consumption OR surface water OR ground water OR stream OR river OR wetlands OR drinking water OR well OR sport fish OR discharge)
Lake Superior AND (health OR health outcomes) AND (environmental stressors OR environmental hazards OR environment OR datasets OR water quality OR air pollution OR air quality OR monitoring OR surveillance OR environmental tracking OR environmental health indicators OR area of concern OR public health tracking OR survey OR toxic OR toxins OR harmful algal blooms OR run-offs OR farm management OR hazardous waste OR contamination OR biomonitoring OR vulnerable populations OR Aboriginal OR Tribal OR children OR pregnant women OR fetus OR diet OR fish consumption OR surface water OR ground water OR stream OR river OR wetlands OR drinking water OR well OR sport fish OR discharge)
Lake Michigan AND (health OR health outcomes) AND (environmental stressors OR environmental hazards OR environment OR datasets OR water quality OR air pollution OR air quality OR monitoring OR surveillance OR environmental tracking OR environmental health indicators OR area of concern OR public health tracking OR survey OR toxic OR toxins OR harmful algal blooms OR run-offs OR farm management OR hazardous waste OR contamination OR biomonitoring OR vulnerable populations OR Aboriginal OR Tribal OR children OR pregnant women OR fetus OR diet OR fish consumption OR surface water OR ground water OR stream OR river OR wetlands OR drinking water OR well OR sport fish OR discharge)

Table S1. *Cont.*

Lake Huron AND (health OR health outcomes) AND (environmental stressors OR environmental hazards OR environment OR datasets OR water quality OR air pollution OR air quality OR monitoring OR surveillance OR environmental tracking OR environmental health indicators OR area of concern OR public health tracking OR survey OR toxic OR toxins OR harmful algal blooms OR run-offs OR farm management OR hazardous waste OR contamination OR biomonitoring OR vulnerable populations OR Aboriginal OR Tribal OR children OR pregnant women OR fetus OR diet OR fish consumption OR surface water OR ground water OR stream OR river OR wetlands OR drinking water OR well OR sport fish OR discharge)
Lake Erie AND (health OR health outcomes) AND (environmental stressors OR environmental hazards OR environment OR datasets OR water quality OR air pollution OR air quality OR monitoring OR surveillance OR environmental tracking OR environmental health indicators OR area of concern OR public health tracking OR survey OR toxic OR toxins OR harmful algal blooms OR run-offs OR farm management OR hazardous waste OR contamination OR biomonitoring OR vulnerable populations OR Aboriginal OR Tribal OR children OR pregnant women OR fetus OR diet OR fish consumption OR surface water OR ground water OR stream OR river OR wetlands OR drinking water OR well OR sport fish OR discharge)

Table S2. Government and Environmental/Health Organization Websites searched.

**Canada**	**United States**
Statistics Canada	Centers for Disease Control and Prevention
Canadian Institutes of Health Information	United States Environmental Protection Agency
Canadian Institutes of Health Research	United States Census Bureau
Institute for Clinical Evaluation Sciences	United States Geological Survey
Environment Canada	United States Food and Drug Administration
Health Canada	United States Department of Agriculture
Public Health Agency of Canada	United States Government
Natural Resources Canada	Agency for Toxic Substances and Disease Registry
Ontario Ministry of Agriculture, Food and Rural Affairs	Michigan Department of Environmental Quality
Ontario Ministry of Health and Long-Term Care	Michigan Department of Community Health
Ontario Ministry of the Environment and Climate Change	Pennsylvania Department of Environmental Protection
Ontario Ministry of Natural Resources	Pennsylvania Department of Conservation and Natural Resources
Ontario Ministry of Northern Development and Mines	Pennsylvania Department of Health
Ontario Ministry of Transportation	Wisconsin Department of Agriculture, Trader and Consumer Protection
Public Health Ontario	Wisconsin Department of Natural Resources
Fisheries and Oceans Canada	Wisconsin Department of Health Services
	Ohio Lake Management Society
	Ohio Department of Health
	Ohio Environmental Protection Agency

Table S2. *Cont.*

Canada	United States
	Ohio Department of Natural Resources
	New York State Department of Environmental Conservation
	New York State Department of Health
	Indiana Department of Environmental Management
	Indiana State Department of Health
	Indiana Geological Survey
	Illinois State Water Survey
	Illinois Environmental Protection Agency
	Illinois Department of Public Health
	Minnesota Pollution Control Agency
	Minnesota Department of Health
	Minnesota Department of Natural Resources
	Minnesota Geological Survey
	California Department of Health Services
	California Environmental Protection Agency

© 2015 by the authors; licensee MDPI, Basel, Switzerland. This article is an open access article distributed under the terms and conditions of the Creative Commons Attribution license (http://creativecommons.org/licenses/by/4.0/).
